# Penetration of the common bile duct into the duodenum by a half-pigtail plastic stent: a rare complication of biliary stenting

**DOI:** 10.1055/a-2645-8893

**Published:** 2025-07-25

**Authors:** Koichi Soga, Suguru Miyo, Fuki Hayakawa, Mayumi Yamaguchi, Masaru Kuwada, Ikuhiro Kobori, Masaya Tamano

**Affiliations:** 1 26263 Department of Gastroenterology, Dokkyo Medical University Saitama Medical Center, Koshigaya, Japan

Endoscopic biliary stenting is a widely accepted intervention for acute cholangitis secondary to choledocholithiasis. Among the various plastic stent designs, pigtail and half-pigtail plastic stents (HPPS) are favored because of their antimigration properties 1 2 . Despite the clinical advantages, rare complications such as stent penetration into adjacent organs may occur, presenting secondary complications 3 . Among these complications, penetration of HPPS through the common bile duct (CBD) into the duodenum is exceedingly rare.

An 85-year-old man presented with fever and pain in the right upper quadrant of the abdomen. Abdominal computed tomography demonstrated emphysematous cholecystitis and a CBD stone. An emergency laparoscopic cholecystectomy was performed. Four days after the emergency operation, an endoscopic procedure was performed for cholecystitis with a CBD stone. An HPPS (7 Fr × 10 cm, Piglet stent; Olympus, Japan) was inserted into the right intrahepatic bile duct ( Fig. 1 ). The procedure was uneventful and the patient’s condition improved.

**Fig. 1:**
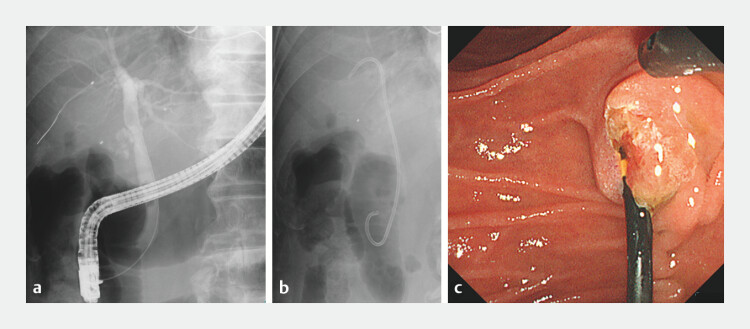
Initial endoscopic retrograde cholangiopancreatography procedure. Plastic stent placement for cholangitis due to common bile duct (CBD) stone. **a** Fluoroscopic image showing the CBD stone. **b** Fluoroscopic image showing a 7 Fr × 10 cm half-pigtail plastic stent (Piglet stent; Olympus, Tokyo, Japan) placed in the right intrahepatic bile duct. **c** Endoscopic view of the duodenal papilla after stent placement.

A second endoscopic procedure was scheduled for 1 month after the cholecystectomy to address the residual CBD stones. During endoscope insertion, the HPPS tip was unexpectedly seen protruding into the duodenal lumen. A comparison with prior imaging confirmed that the previously placed HPPS had shifted distally. Fluoroscopic cholangiography also showed that the shaft of the stent had penetrated through the CBD wall into the duodenum ( Fig. 2 ). The stent was gently extracted using grasping forceps. The procedure for extracting the remaining CBD stones was successful, and the patient had no further complications thereafter ( Fig. 3 , Video 1 ).

**Fig. 2:**
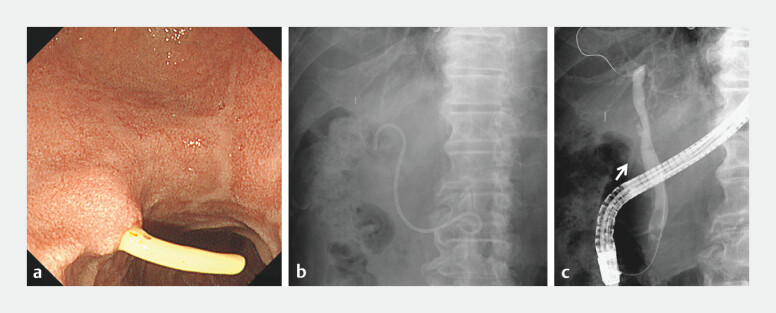
Penetration of the half-pigtail plastic stent (HPPS) from the common bile duct (CBD) into the duodenum. **a** Endoscopic image revealing the distal tip of the HPPS protruding into the lumen of the duodenum. **b** Abdominal X-ray showing penetration of the HPPS from the CBD into the duodenum. **c** Fluoroscopic cholangiogram showing contrast leaking (allowed) from the CBD directly into the duodenal lumen through the path of the stent.

**Fig. 3:**
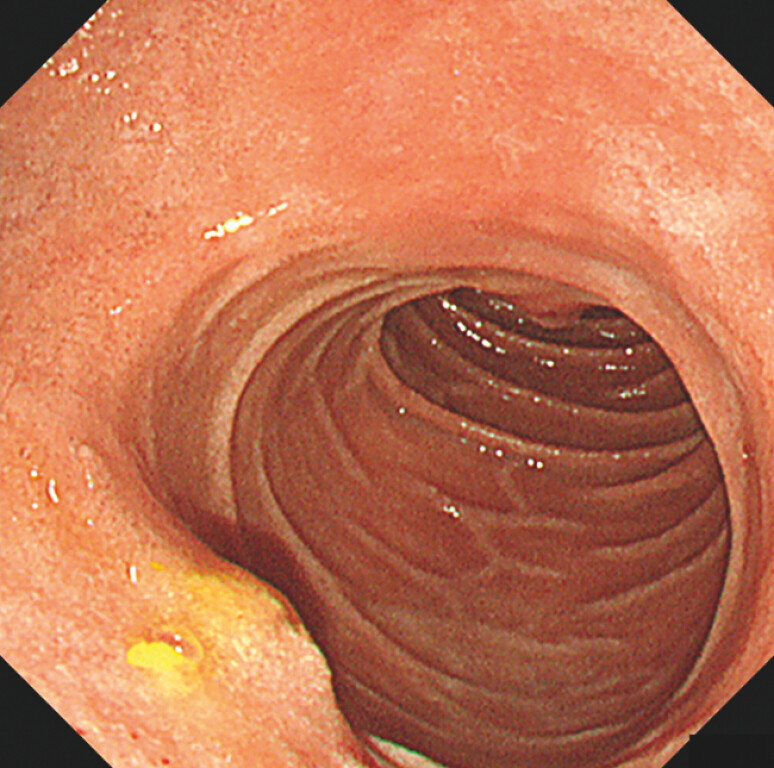
Stent removal and post-extraction appearance. Endoscopic image of the duodenal wall after removal revealed a small fistulous opening without bleeding.

Penetration of the common bile duct into the duodenum by a half-pigtail plastic stent.Video 1

Several mechanisms may underlie such events, including sustained mechanical pressure and the force vector transmitted by the pigtail structure toward the duodenum. This case report highlights a rare but significant adverse event associated with HPPS. Even with an anatomically favorable placement, penetration may occur over time, necessitating careful follow-up and stent removal strategies.

Endoscopy_UCTN_Code_CPL_1AK_2AI

## Notes

Endoscopy E-Videos https://eref.thieme.de/e-videos
*E-Videos* is an open access online section of the journal *Endoscopy* , reporting on interesting cases and new techniques in gastroenterological endoscopy. All papers include a high-quality video and are published with a Creative Commons CC-BY license. Endoscopy E-Videos qualify for HINARI discounts and waivers and eligibility is automatically checked during the submission process. We grant 100% waivers to articles whose corresponding authors are based in Group A countries and 50% waivers to those who are based in Group B countries as classified by Research4Life (see: https://www.research4life.org/access/eligibility/ ). This section has its own submission website at https://mc.manuscriptcentral.com/e-videos .
